# Combination of Modern Radiotherapy and New Targeted Treatments for Breast Cancer Management

**DOI:** 10.3390/cancers13246358

**Published:** 2021-12-18

**Authors:** Arnaud Beddok, Paul Cottu, Alain Fourquet, Youlia Kirova

**Affiliations:** 1 Department of Radiation Oncology, Institut Curie, 75005 Paris, France; alain.fourquet@curie.fr (A.F.); youlia.kirova@curie.fr (Y.K.); 2 Department of Radiation Oncology, Institut Curie, 91400 Orsay, France; 3 Laboratory of Translational Imaging in Oncology (LITO), UMR (U1288), Institut Curie, 91400 Orsay, France; 4 Department of Medical Oncology, Institut Curie, 75005 Paris, France; paul.cottu@curie.fr

**Keywords:** multimodal treatment, radiation tolerance, drug tolerance

## Abstract

**Simple Summary:**

Since the introduction of hormone therapy for the treatment of breast cancer (BC) three decades ago, many new targeted therapies have been developed. Some of them are currently used, such as HER2 inhibitors, while others are still under development, such as cell cycle (CDK) inhibitors, immune checkpoint (PD1/PDL1) inhibitors, or molecules acting on DNA damage (PARP) repair. Besides this, radiation therapy (RT) is commonly used either as adjuvant treatment for early BC after breast conservative surgery or in palliative intent for the treatment of metastatic sites. Our research has shown that the combinations of the most commonly used targeted treatments and RT were feasible with a few toxicities. Nevertheless, most of the knowledge on this subject is based on retrospective studies and a small number of patients and care should be taken in this setting until these results would be confirmed in prospective randomized studies.

**Abstract:**

Background: The objective of the present study was to review the essential knowledge about the combinations of the most commonly used or under development targeted treatments and radiation therapy (RT). Methods: Preclinical and clinical studies investigating this combination were extensively reviewed. Results: Several studies showed that the combination of RT and tamoxifen increased the risk of radiation-induced pulmonary toxicity; therefore, both modalities should not be given concomitantly. The combination of HER2 inhibitors (trastuzumab, pertuzumab) and RT seems to be safe. However, trastuzumab emtansine (T-DM1) should not be administered concurrently with brain RT since this combination could increase the risk of brain radionecrosis. The combination of RT and other new target treatments such as selective estrogen receptor degradants, lapatinib, cell cycle inhibitors, immune checkpoint inhibitors, or molecules acting on DNA damage repair seems feasible but was essentially evaluated on retrospective or prospective studies with a small number of patients. Furthermore, there is considerable heterogeneity among these studies regarding the dose and fractionation of radiation, the dosage of drugs, and the sequence of treatments used. Conclusions: The combination of RT with most targeted therapies for BC appears to be well-tolerated, but these results need to be confirmed in prospective randomized studies.

## 1. Introduction

**Figure 1:**
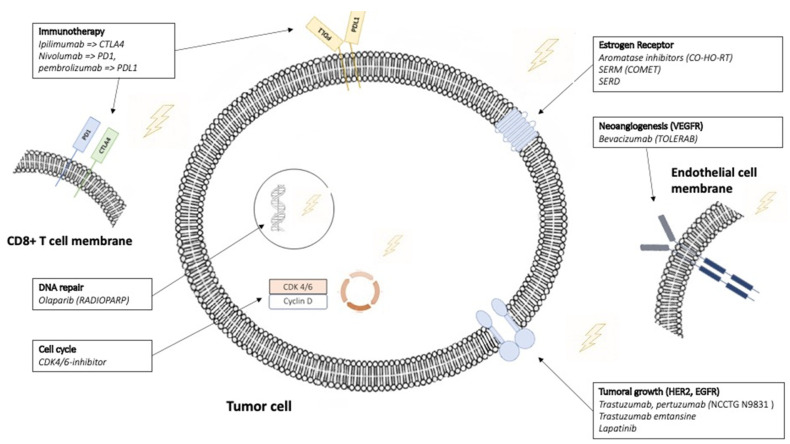
Targeted treatments evaluated in combination with radiotherapy. This figure illustrates the six target types of treatments for which combination with radiation therapy was described in this review. HER2: Human Epidermal growth factor Receptor 2, EGFR: Epidermal growth factor Receptor.

Radiation therapy (RT) is one of the most common therapeutic modalities, along with surgery and systemic treatment, for the treatment of breast cancer (BC). Many studies have demonstrated the interest of RT as adjuvant treatment for early BC after breast conservative surgery [1]. RT is also currently used in palliative intent for the treatment of metastatic sites. In both contexts, RT is potentially combined with several different drugs. In this situation, the question is often asked whether these two treatments can be combined concomitantly or whether the systemic treatment should be suspended during RT. The objective of the present study was to review the essential knowledge about the combinations of the most commonly used targeted treatments and RT (Figure 1). For each molecule, we emphasize the safety and the new developments of this combination.

## 2. Materials and Methods

References for this review were identified through searches of PubMed with the search terms “tamoxifen AND radiotherapy”, “aromatase inhibitor and radiotherapy”, “SERD and radiotherapy”, “Human Epidermal growth factor Receptor 2-inhibitor and radiotherapy”, “Vascular endothelial growth factor inhibitor and radiotherapy”, “immunotherapy and radiotherapy”, “CDK inhibitor and radiotherapy”, and “Olaparib and radiotherapy” from 1990 until June 2021. Articles were also identified through searches of the authors’ own files. Only papers published in English were reviewed. The final reference list was generated on the basis of originality and relevance to the broad scope of this Review. We also conducted research on the https://clinicaltrials.gov/ct2/home (accessed on 13 December 2021) website to assess the ongoing randomized trials about the combination of RT and targeted treatments in BC (Table 1).

## 3. Results

### 3.1. Hormonotherapy

The inhibition of the estrogen receptor (ER) pathway is currently one of the main treatment strategies for ER-positive breast cancer (BC). The three main methods available to target the estrogenic pathway are: aromatase and GnRH (gonadotropin hormone analogues) inhibitors, which induce a decrease in the endogenous production of estradiol; the selective estrogen receptor modulators (SERMs), which bind directly to estrogen; and the selective estrogen receptor degradants (SERDs), which induce estrogen receptor degradation [2]. 

Several pre-clinical and retrospective clinical studies have suggested that the concomitant and/or sequential combination of tamoxifen and breast irradiation could induce a risk of pulmonary fibrosis (PF) [3]. The key mechanism explaining the increase PF when combining tamoxifen and RT would be via the induction of TGF-β synthesis. In 1992, Butta et al. [4] studied the effect of tamoxifen on the regulation of TGFβ in vivo. Three months after tamoxifen treatment, a dramatic increase in the extracellular TGF-β1 was detected in repeated biopsies in all 10 studied patients. In 2013, Yavas et al. [5] confirmed this result when they compared in vivo the effect of aromatase inhibitors (anastrozole and letrozole) and tamoxifen on radiation-induced PF. Eighty female Wistar albino rats were divided into several groups to compare RT alone, the combination of RT and tamoxifen, and the combination of RT and aromatase inhibitor. A single dose of 12 Gy RT was given to both lungs. Tamoxifen, anastrozole, and letrozole were started 1 week before the RT and continued until the animals were sacrificed 16 weeks after the RT. When compared with the RT only group, the concomitant RT and tamoxifen group increased the radiation-induced pulmonary fibrosis (*p* = 0.005). In a prospective randomized study, Bentzen et al. (1996) [6] examined the effect of tamoxifen on the incidence of PF in patients treated with mastectomy and adjuvant RT with or without concomitant tamoxifen. They found a statistically significant two-fold increase in PF if the patients had been receiving tamoxifen. Several other prospective studies have shown that tamoxifen was an independent risk factor of PF when combined with RT [7,8]. However, two other retrospective clinical studies did not identify any differences between the concomitant or sequential use of tamoxifen for the risk of PF [9,10]. A randomized trial is currently ongoing to compare the risk of radiation-induced lung toxicity among patients receiving tamoxifen concurrently or sequentially with RT (NCT00896155, accessed on date 2021 December) [11]. For now, there is more evidence to suggest that the combination of RT and tamoxifen increases the risk of radiation-induced pulmonary toxicity and that they should not be given concomitantly.

Moreover, there is clinical evidence that five years of adjuvant aromatase inhibitor (AI) improves recurrence-free survival in postmenopausal early BC patients. Indeed, results from the ATAC trial [12] demonstrated that recurrence rates remained significantly lower on anastrozole compared with tamoxifen. In 2007, Bollet et al. [13] have reported that AI and RT could be given concurrently to post-menopausal patients with both acceptable safety and good efficacy. The safety of AI and concurrent adjuvant radiotherapy was also shown in the CO-HO-RT trial, a prospective randomized phase 2 study [14]. The authors found no increase in cutaneous toxicity in BC patients receiving letrozole and concurrent normofractionated breast RT, delivering 2 Gy per daily fraction. Concurrent AI did not decrease the efficacy of irradiation at a median follow-up of 26 months. Moreover, Chargari et al. [15] (2012) evaluated the toxicity of concurrent use of AI and hypofractionated RT in 19 patients. At the end of the RT course, acute toxicity was low in most patients, with only 1 grade 3 cutaneous toxicity. Besides this, AI did not increase the risk of radiation-induced toxicity [5]. Although this is not common practice in France, these results suggest that aromatase inhibitors could be started during RT. This was done and recommended during the COVID-19 period to avoid delaying the start of treatment for patients [16].

Among the SERD, fulvestrant is unique amongst currently approved ER ligand therapeutics due to its classification as a full ER antagonist [17]. However, the full clinical potential of fulvestrant is thought to be limited by its poor physicochemical properties and exposure limitations due to its administration by intramuscular injection. Metcalfe et al. (Abstract P5-04-07 SABCS 2019) recently described for the first time GDC-9545 which, like fulvestrant, consistently induced ER turnover and drove deep transcriptional suppression of ER, resulting in a robust in vitro anti-proliferative activity [18]. The in vivo efficacy of GDC-9545 in this model was greater than fulvestrant at clinically relevant exposures. Currently, two phase I-II clinical trials are evaluating the outcomes and toxicities of this new drug in locally advanced or metastatic ER + BC patients. NCT03916744 (accessed on date 2021 December) is evaluating the pharmacodynamics, pharmacokinetics, safety, and biologic activity of GDC-9545 in participants with Stage I-III operable ER +, HER2 -, untreated BC. NCT03332797 (accessed on date 2021 December) is evaluating the safety, pharmacokinetic (PK), pharmacodynamic (PD) activity, and preliminary anti-tumor activity of GDC-9545 as a single agent and in combination with palbociclib and/or luteinizing hormone−releasing hormone (LHRH) agonist in participants with advanced or metastatic ER +, HER2 -, BC. Several other oral SERD (such as Elacestrant (RAD1901) [19,20], AZD-9496, and LSZ102) are in clinical development [21]. The combination of RT and these molecules has not yet been evaluated. 

### 3.2. Target the Tumor Growth: HER-2 Inhibitor

Currently, four molecules targeting the Human Epidermal growth factor Receptor 2 (HER2 receptor) are commonly used in the management of patients with BC, mainly at the metastatic stage: trastuzumab, pertuzumab, lapatinib, and T-DM1. 

Trastuzumab and pertuzumab are both humanized monoclonal antibodies, directed against the extracellular domain of the receptor. This induces blocking the MAP kinase signaling pathways and the PI3K-Akt pathway, which slows the cell cycle and decreases tumor cell proliferation [22]. Double blockage consists of the concurrent administration of Trastuzumab and Pertuzumab. Several preclinical studies using BC models have shown that anti-HER2 therapy could act as a radiosensitizer [23,24]. In particular, Hou et al. recently conducted in vitro and in vivo studies to investigate whether HER-2 overexpression was associated with radiosensitivity in BC [24]. They found that HER-2 reduced radiosensitivity in two BC cell lines, MCF-7 (low HER2 expression) and MDA-MB-231 (HER2 is not expressed). Moreover, animal experiment results showed HER2 could enhance the radioresistance of xenograft tumors. For early BC management, sequential anthracycline and taxanes administered concurrently with trastuzumab or docetaxel, carboplatin, and trastuzumab for six cycles are recommended in high-risk HER2-positive disease. An alternative regimen in a lower-risk, node-negative, HER2-positive population is paclitaxel and trastuzumab [25]. Considering these indications, trastuzumab is frequently administered concomitantly with locoregional radiotherapy. The main issue in the combination of an HER inhibitor and locoregional breast RT would be the risk of cumulative cardiac toxicity. Indeed, it is known that independently, HER2 blockade on the one hand [26] and locoregional RT for BC [27], especially in case of breast node irradiation, on the other hand, could induce cardiac toxicity. Several retrospective and prospective studies have evaluated the skin and cardiac toxicity of this combination (Table 1 [28,29,30,31,32]). The ranges of ≥ Grade 2 cardiac, skin, and esophagitis toxicity were 0–25.8%, 2.9–30.9%, and 1.6–12%, respectively. In 2009, Halyard et al. carried out an ancillary study of the NCCTG Phase III Trial N9831 study in which they investigated the acute and late safety of this combination in 982 patients treated for BC (adjuvant treatment) [30]. No significant differences among arms were found in the incidence of acute skin reaction, pneumonitis, dyspnea, cough, dysphagia, or neutropenia. At a median follow-up of 3.7 years, RT with trastuzumab did not increase the relative frequency of cardiac events regardless of the treatment side. Several more recent retrospective studies reported the good safety of the combination of RT and dual HER2 receptor inhibitor [33,34]. In particular, Aboudaram et al. have recently shown that the administration of dual HER2 blockade in 55 patients receiving locoregional RT was safe in terms of skin, gastrointestinal, and general toxicity [31]. No significant cardiac toxicity was observed, apart from a slight decrease in left ventricular ejection fraction (LVEF), which is expected during the HER2 blockade [26]. Besides this, as these treatments are used in patients with metastatic BC, it is also important to know the tolerance of the combination HER2 inhibitor and RT for brain metastases (BM) [35]. Chargari et al. (2011) have shown in 31 patients low toxicity of trastuzumab concurrently with whole-brain radiation therapy (WBRT) [36]. To our knowledge, no study has yet tested the combination of trastuzumab and stereotactic radiotherapy (SBRT). Based on these results, it appears that trastuzumab and pertuzumab could be administered concurrently with radiotherapy, either regionally or at metastatic sites (such as the brain). To our knowledge, no prospective or randomized clinical randomized trials are currently ongoing on this subject. 

Trastuzumab emtansine (T-DM1) is an antibody–drug conjugate currently approved as monotherapy for the second-line treatment for HER2-positive metastatic BC pretreated by trastuzumab and taxanes [37]. Two recent studies reported that the combination of SBRT and TDM1 in patients with BM was possible but with an increased risk of brain radionecrosis [38,39]. Two randomized trials (NCT02135159, NCT03190967, accessed on date 2021 December) are currently ongoing on this subject and probably will allow one to specify the best appropriate time between treatments. Given these results, TDM1 should not be administered concurrently with brain radiotherapy, before the results of both clinical trials. Moreover, since the KATHERINE trial results [40], patients with HER2-positive BC with pathologic invasive residual disease at surgery after standard preoperative chemotherapy and HER2-targeted therapy should be offered 14 cycles of adjuvant TDM1 [41]. In a recent study, Mignot et al. [42] have evaluated the in vitro effects of T-DM1 and concurrent irradiation on HER2-positive BC cells. Their results indicated that T-DM1 was not a radiation-sensitizer, assessing cell survival. To our knowledge, no in vivo study was currently published on this subject. Zolcsák et al. have also recently reported in a retrospective study of 14 patients that TDM-1 concomitantly administered with locoregional RT was well supported, without high-grade cardiac toxicity [43]. Of course, these results were obtained using a small number of subjects, and a validation cohort would be necessary to confirm these results. In the meantime, it would be preferable not to combine locoregional radiotherapy and TDM1 concurrently.

Lapatinib is a reversible dual tyrosine kinase inhibitor (TKI) targeting the epidermal growth factor (EGFR, ErbB-1) and HER2 (ErbB-2). Several preclinical studies using BC models have shown that lapatinib could be a radiosensitizer. Yu et al. (1996) conducted an in vitro experiment using SKBR3 and BT474 breast carcinoma cells exhibiting HER2/neu amplification. Pretreatment of lapatinib increased the radiosensitivity of both BC cells lines. Moreover, Sambade et al. (2010) demonstrated that lapatinib could radiosensitize HER2+ SUM225 BC xenografts [44]. Lapatinib has demonstrated effectiveness in BM from HER2-overexpressing BC. WBRT in association with lapatinib was well tolerated in a cohort of 21 patients with BM from BC [45]. In a retrospective cohort study, lapatinib in combination with SBRT significantly increased intracranial complete response rate (35% versus 11%) [46]. Concurrent lapatinib was not associated with an increased risk of grade 2+ radiation necrosis (1.0% with concurrent lapatinib vs. 3.5% without, *p* = 0.27). Several prospective phase 2 cohorts (NCT00470847, NCT01622868, NCT00379509, accessed on date 2021 December) are ongoing to evaluate the tolerance and the outcomes of a combination of lapatinib and WBRT. To our knowledge, there are currently no published results about the combination of locoregional RT and lapatinib. A prospective phase 2 cohort (NCT01868503, accessed on date 2021 December) is ongoing to evaluate the tolerance and the outcomes of a combination of lapatinib and locoregional RT. Several other clinical studies such as NCT04582968 or NCT01494662 (accessed on date 2021 December), are ongoing and will allow assessing the safety and the efficacy of RT combined with respectively pyrotinib and neratinib, for patients with BM from BC. The published results were obtained using a small number of subjects. In the attempt of the results of mentioned clinical trials, it would be preferable not to combine RT and lapatinib concurrently.

### 3.3. Target the Tumor Angiogenesis: VEGF-Inhibitor

Bevacizumab is a VEGF-targeting monoclonal antibody. Several clinical trials demonstrated the interest of bevacizumab for improving progression-free survival and response in patients with advanced or metastatic BC [47,48]. From these studies, only limited data were available concerning the safety of the combination of bevacizumab and locoregional or palliative radiotherapy. However, several preclinical studies with different models have shown that VEGF inhibitor could have a radiosensitizing effect when it is combined with radiotherapy (RT) [49,50]. As for the other molecules presented in this review, a radiosensitizing effect could be useful for the anti-tumor efficacy of radiotherapy but could be responsible for an increase in radiation-induced toxicities. In 2011, Goyal et al. [51] reported the outcomes and toxicity of the first fourteen patients treated with the combination of bevacizumab and locoregional RT for BC. None of the patients receiving bevacizumab plus RT experienced ≥ Grade 3 toxicity within the irradiated volume. Moreover, from October 2007 to August 2010, the French multicenter non-interventional observational cohort TOLERAB study included patients with non-metastatic BC treated by local or locoregional radiotherapy and concurrent bevacizumab after either neoadjuvant or adjuvant chemotherapy in the trials BEVERLY 1, BEVERLY 2, BEATRICE, or BETH [52]. Acute and early late toxicities were acceptable: Grade 3 acute radiation dermatitis was observed for only four patients, none patients had grade 3 pneumonitis or esophagitis. Clement Zhao et al. [53] recently reported the 5-years outcomes of 46 patients included in this prospective study. Only eight patients reported late toxicity, none grade 3. For the few patients currently being treated with bevacizumab for localized BC, bevacizumab may be administered concomitantly with RT. The A-Plus trial (NCT02185352, accessed on date 2021 December) is currently ongoing to assess to outcomes and toxicity of the combination of bevacizumab and WBRT.

### 3.4. Immunotherapy

Among the many different types of immunotherapies currently being studied, immune checkpoint inhibitors (ICI) are the most employed. Ipilimumab is an anti-CTLA-4 antibody and Nivolumab and pembrolizumab act on the PD-1/PD-L1. These treatments are designed to restore the capacity of the immune system to recognize and eliminate a tumor. Several preclinical studies have investigated the combination of an ICI and RT. In 2005, Demaria et al. [54] have in particular showed that when a poorly immunogenic murine model of metastatic BC (4T1 cells) was implanted subcutaneously in mice, the combination of 9H10 (an anti- CTLA-4 antibody) and RT was the only regimen which allowed an improvement of survival and especially CTL-mediated inhibition of the formation of lung metastases. Verbrugge et al. [55] also showed that the antitumor effects of RT, in established triple-negative breast tumors could be enhanced in particular with anti (PD)-1 antibody. One explanation for these results could be the upregulation of PD-L1 in the tumor microenvironment after RT [56]. In a recent study, Ho et al. (2019) have for the first time prospectively studied pembrolizumab in combination with RT in patients with triple-negative BC with at least two evaluable lesions [57]. The response rates of non-irradiated lesions in the three patients with partial response were 60%, 54%, and 34%, and these responses were lasting (31, 21, and 40 weeks). In 2020, Barroso-Sousa et al. [58] performed a prospective study to investigate whether combining pembrolizumab with palliative RT improves outcomes in patients with hormone receptor-positive metastatic BC. Unfortunately, there were no objective responses, and the study was closed before the scheduled end of recruitment. Several prospective studies on the combination of ICI and RT for BC treatment are currently ongoing (see Table 2 of [59]). It should be noted that only a few data are currently available concerning the toxicity of combined ICI and RT [60], and for the moment, the combination of both treatments should be reserved for clinical trials.

### 3.5. Target the Cell Cycle: CDK-Inhibitor

In 2016, the phase 3 study PALOMA-2 showed that the addition of a CDK inhibitor to standard endocrine therapy significantly improved outcomes in the first-line treatment of ER-positive, HER2- negative advanced BC [61,62]. The development of palbociclib for the treatment of hormone-receptor-positive advanced BC was based on the findings of preclinical studies that identified a dependence of hormone receptor-positive BC on CDK signaling and a synergistic effect from targeting the ER, cyclin-D–CDK4/6–Rb pathway [63]. Several pre-clinical studies have shown that the combination of RT and palbociclib could increase the anti-tumor effect in brain cancer cell lines [64,65,66]. However, the clinical effects of combining palbociclib and RT is least well-known. Several small retrospective studies have reported that the combination of RT and CDK inhibitor was generally well tolerated [67]. The series mainly reported the follow-up of patients irradiated on metastatic lesions. In 2017, Hans et al. [68] reported in a short letter very preliminary results of five patients treated with this combination and did not observe increased toxicity, particularly hematological toxicity. Conversely, in a recent case report, Kawamoto et al. observed severe acute radiation-induced enterocolitis after combined palbociclib and palliative RT [69]. Another more recent study retrospectively evaluated the toxicity and the outcomes of 16 women who received palliative RT in close temporal proximity to palbociclib administration [70]. The following sites were irradiated in decreasing order of frequency: bone, brain, and mediastinum. Most of the hematologic toxicities were grade 1, and no acute or late > grade 2 skin, neurologic, or gastrointestinal toxicities were noted. Therefore, the consequences of a such combination remain unknown and most radiation oncologists prefer to suspend the CDK-I during radiotherapy.

Although the use of CDK4/6 inhibitors is currently limited to the metastatic setting, there are ongoing efforts to evaluate the efficacy of CDK4/6 inhibitors in the upfront setting for women with locally advanced or high-risk ER+ disease [71]. In contrast to the conventional use of CDK4/6 inhibitors in the metastatic setting, Pesh et al. recently studied the effects of CDKi and radiation (RT) in multiple preclinical BC models [72]. They demonstrated that short-term treatment of ER+ BC cell lines with the CDK4/6 inhibitors palbociclib, ribociclib, and abemaciclib led to alterations in many cellular pathways, including suppression of cell cycle signaling and changes in the DNA damage response. In ER+ BC cells that are sensitive to CDK4/6 inhibitor monotherapy, the combination of CDK4/6 inhibition and ionizing RT led to significant radiosensitization with each of the three clinically approved CDK4/6 inhibitors. Petroni et al. [73] recently reported that radiotherapy given before CDK4/6 inhibitors produces superior antineoplastic effects compared with other therapeutic schedules. In a study published in 2020, authors reported the outcomes of nine patients with de novo metastatic breast carcinoma, who were at least stable after 6 months of palbociclib, received LR irradiation and palbociclib [74]. Palbociclib had to be discontinued during RT in two patients because of grade 3 dermatitis and pain, and grade 2 esophagitis, respectively. However, in the first case, the patient had undergone bilateral mastectomy and axillary lymph node dissection for very advanced LR disease before RT. This case was the only case in which the CTV included the bilateral chest wall and all bilateral regional lymph node areas. Moreover, in both cases, the PTV was particularly large compared to other LR irradiation volumes. It is therefore difficult to conclude that the combination of palbociclib and RT was responsible for these toxicities.

Currently, two ongoing clinical trials are testing the combination of palbociclib and RT. The ongoing prospective phase 2 ASPIRE (NCT03691493, accessed on date 2021 December) trial is assessing RT combined with palbociclib and hormone therapy for bone metastases in BC patients whereas the PALATINE (NCT03870919, accessed on date 2021 December) prospective trial will assess local breast treatment, surgery and/or RT in advanced BC.

### 3.6. Target the DNA Repair: PARP-Inhibitor

Poly-(adenosine diphosphate-ribose)-polymerase (PARP) is a family of enzymes involved in DNA replication, transcription, repair, and cell death. In 2005, two studies showed that dysfunction of homologous recombination such as in BRCA1 and BRCA2 mutated BC cells triggered a high sensitization to PARP-inhibitor [75,76]. The targeted treatments have also many qualities required for radiosensitizing effects [77]. Associated with PARP-inhibitor, RT would induce DNA damages, such as double-strand break, which could not be repaired, in particular in BRCA mutated [78]. Nevertheless, several studies demonstrated that radiosensitization was independent of BC intrinsic subtype and did not appear to be related to the BRCA1 mutation status [79,80]. In 2015, Jagsi et al. reported the toxicity data of a phase I study which included 30 patients receiving locoregional radiotherapy for local recurrence of Triple-negative BC, and PARP-inhibitor (Veliparib). Initially, the combination was well tolerated with only one grade 4 radiation-induced dermatitis [81]. However, the grade 3 toxicity rate was 46.7% at year 3 [82]. More recently, Loap et al. reported the first results of RADIOPARP (NCT03109080, accessed on date 2021 December), a phase 1 trial that evaluated the combination of Olaparib and RT for 24 patients with triple-negative BC [83]. At a 1-year follow-up, no treatment-related grade ≥3 toxicity was observed [84]. One patient (4.2%) had persistent grade 2 adverse events (breast pain, fibrosis, and deformity). Moreover, a phase 2 trial evaluating olaparib with RT for inflammatory TNBC is currently recruiting (NCT03598257, accessed on date 2021 December) and other PARP-inhibitors are under evaluation in phase 1 trials: rucaparib (NCT03542175, accessed on date 2021 December) and Niraparib (NCT03945721, accessed on date 2021 December). Although these results are very encouraging, it seems preferable to wait for the results of these prospective studies before routinely administering PARP-inhibitors and RT concurrently.

The Table 3 summarizes the main results of preclinical and clinical studies that evaluated the safety of the concomitant combination of radiotherapy and targeted therapy.

## 4. Discussion

In the present study, we have performed a comprehensive synthesis of knowledge on the combination of radiotherapy and targeted therapies currently used for the treatment of localized or metastatic breast cancer (BC).

It is important to note that more and more prospective studies are underway to investigate these combinations. However, the number of randomized trials is very limited (Table 1), and it would certainly be useful to propose a multicenter, randomized trial for each molecule to answer the question of the toxicity of these combinations. This is particularly important because the comparison of trials is not always obvious. Indeed, there is considerable heterogeneity among the reported studies regarding the dose and fractionation of radiation, the dosage of drugs, and the sequence of treatments used. This can lead to contradictory results as we have seen for tamoxifen and the risk of pulmonary fibrosis.

The study of the combination of new treatments with radiotherapy is complex because of the many factors involved. Concerning RT, dose and fractionation may in themselves have an impact on the outcome of a combination. It is particularly right for immunotherapy. In a murine model of weakly immunogenic BC (TSA cell line), Dewan et al. showed that the best abscopal effect was obtained with a dose of 24 Gy in 3 fractions of 8 Gy (vs 1. 20 Gy and 5.6 Gy) and when the anti-CTLA-4 antibody was administered over the days following irradiation, without exceeding a 4-day interval, beyond which the benefit of this combined treatment was no longer observed [85].

## 5. Conclusions

In conclusion, a large number of molecules continue to emerge and, in this context, care should be taken. For molecules for which there is sufficient pre-clinical and clinical scientific evidence, the concomitant association of locoregional or metastatic radiotherapy with this molecule seems feasible. For the others, it is necessary to wait for the results of clinical trials before allowing this type of combination.

## Figures and Tables

**Table 1:** Main ongoing randomized clinical trials testing the combination of targeted treatments and RT for breast cancer treatment (accessed on date 2021 December)

Target	Name	Number	Recruitment Status	Endpoint
Estrogen receptor (ER)				
Tamoxifen + locoregional RT	CONSET trial	NCT00896155	Unknown	Pulmonary fibrosis
Tumor growth				
Trastuzumab Emtansine (T-DM1) + brain RT	BIRTH trial	NCT02135159	Completed	Brain radionecrosis
Tumor angiogenesis				
Bevacizumab + brain RT	A-Plus	NCT02185352	Active, not recruiting	Brain-specific progression free survival
Cell cycle				
Palbociclib + locoregional RT	PALATINE	NCT03870919	Recruiting	Overall survival
DNA repair				
Olaparib +/− locoregional RT		NCT03598257	Recruiting	Invasive Disease-Free Survival

**Table 2:** Main studies investigated the combination of HER2 inhibitor and locoregional breast cancer radiation therapy.

Studies	Prospective/ Retrospective	Number of Patients	Mono or Double HER2 Blockage	IMC Irradiation	Anthracyclines	≥Grade 2 Cardiac Toxicity	≥Grade 2 Skin Toxicity	≥Grade 2 Esophagitis
Belkacémi et al. 2008 [32]	Prospective	146	Trastuzumab	76%	NA	10%	51%	12%
Halyard et al. 2009 [30]	Prospective	982	Trastuzumab	0%	100%	2.1%	6.2%	NA
Caussa et al. 2011 [29]	Prospective	106	Trastuzumab	83%	92%	5.7%	15.1%	3.8%
Jacob et al. 2014 [28]	Prospective	308	Trastuzumab	73.7%	90.9%	25.8%	2.9%	1.6%
Aboudaram et al. 2021 [31]	Retrospective	55	Trastuzumab-Pertuzumab	NA	NA	0%	30.9%	1.8%

IMC: internal mammary chain.

**Table 3:** Main preclinical and clinical published results of the combination of targeted treatments and radiation therapy.

Targeted Treatments	Main Preclinical Results	Ref.	Main Clinical Results	Ref.
**Estrogen receptor**				
SERM (Tamoxifen)	In in vivo model: high frequency of RIPF in concurrent administration of tamoxifen and RT	[5]	In retrospective and prospetive studies: high frequency of RIPF in concurrent administration of tamoxifen and RT	[6,7,8]
Aromatase Inhibitor	In in vivo model: low frequency of RIPF in concurrent administration of AI and RT	[5]	In retrospective studies: low frequency of any grade 3 toxicity (incuding RIPF)	[13,14,15]
**HER2-inhibitor**				
Tastuzumab, Pertuzumab (both humanized monoclonal antibodies, directed against the extracellular domain of the receptor)	In in vitro models: HER2 reduces breast cancer celles radiosensitivity	[23,24]	In retrospective and prospetive studies: low frequency of cardiac toxicity in concurrent administration of HER2-inhibitor and locoregional RT and low frequency of brain toxicity in concurrent administration of HER2-inhibitor and brain RT	[28,29,30,31,32,36]
T-DM1 (antibody–drug conjugate)	In in vitro models: T-DM1 was not a radiation-sensitizer on HER2-positive breast cancer cells	[42]	In retrospective studie: high frequency of brain radionecrosis in concurrent administration of T-DM1 and brain SBRT, low frequency of cardiac toxicity in concurrent administration of T-DM1 and locoregional RT	[38,39,43]
Lapatinib, pyrotinib * and neratinib * (tyrosine kinase inhibitor targeting the epidermal growth factor (EGFR, ErbB-1) and HER2 (ErbB-2))	In in vitro and in vivo models: lapatinib increased the radiosensitivity of BC cells lines and BC xenografts	[44,85]	In retrospective studie: low frequency of brain radionecrosis in concurrent administration of lapatinib and brain RT	[45,46]
**VEGFR inhibitor**				
Bevacizumab	In in vitro and in vivo models: VEGF inhibitor has a radiosensitizing effect when it is combined with RT	[49,50]	In retrospective studies; low frequency of any grade 3 toxicity in concurrent administration of bevacizumab and RT	[51,53]
**Immunotherapy**				
Ipilimumab (anti-CTLA-4 antibody)	In in vivo model: Increase overall survival in immunogenic murine model of metastatic BC	[54]	N/A	
Nivolumab and pembrolizumab (antibody PD-1/PD-L1 antibody)	In in vivo model: Antitumor effects of concurrent administration of RT and nivolumab in established triple-negative breast tumors	[55]	In prospective study: outcomes improvements in concurrent administration of pertuzumab and RT for metastatic BC, no data for toxicity	[57,58]
**CDK inhibitor**				
Palbociclib, ribociclib, abemaciclib (tyrosine kinase inhibitor targeting CDK4/6 cyclin D)	In in vitro and in vivo models: Coucurrent administration of CDK4/6 inhibition and RT led to significant radiosensitization in multiple BC models	[72,73]	In retrospective studies: several cases of grade 3 toxicity	[69,70,74]
**PARP-inhibitor**				
Olaparib, velaparib, rucaparib *, niraparib *	In in vitro study: Concurrent administration of PARPi and RT induce more DNA damages in particular in BRCA mutated BC cell lines	[80]	In retrospective and prospective studies: high frequency of late toxicity with velaparib, low frequence of late toxicity with olaparib	[82,84]

In this table, we reported the main preclinical and clinical published results of the combination of targeted treatments and radiation therapy. The targets are in bold. Abbreviations: SERM: Selective estrogen receptor modulators, RIPF: radiation-induced pulmonary fibrosis, RT: radiation therapy, AI: aromatase inhibitor, HER2-inhibitor: Human Epidermal growth factor Receptor 2-inhibitor, EGFR: Epidermal growth factor Receptor, T-DM1: Trastuzumab emtansine, SBRT: stereotactic body radiation therapy, VEGFR-inhibitor: Vascular endothelial growth factor receptor inhibitor, BC: breast cancer, PARP-inhibitor: Poly-(adenosine diphosphate-ribose)-polymerase inhibitor. *: studies currently ongoing.

## Abbreviations

ADC	antibody–drug conjugate
BC	breast cancer
BM	brain metastases
CDK-I	CDK inhibitor
EGFR	epidermal growth factor
ER	estrogen receptor
GnRH	gonadotropin hormone analogues
ICI	immune checkpoint inhibitors
LHRH	luteinizing hormone−releasing hormone
LVEF	Left ventricular ejection fraction
PD	pharmacodynamic
PF	pulmonary fibrosis
PK	pharmacokinetic
RT	radiation therapy
SBRT	stereotactic body RT
SERD	selective estrogen receptor degradants
SERM	selective estrogen receptor modulators
T-DM1	Trastuzumab emtansine
TKI	tyrosine kinase inhibitor
WBRT	whole-brain RT
